# PONDx: real-life utilization and decision impact of the 21-gene assay on clinical practice in Italy

**DOI:** 10.1038/s41523-021-00246-4

**Published:** 2021-05-05

**Authors:** Francesco Cognetti, Riccardo Masetti, Alessandra Fabi, Giulia Bianchi, Donatella Santini, Alessia Rognone, Giovanna Catania, Domenico Angelucci, Giuseppe Naso, Mario Giuliano, Lucia Vassalli, Patrizia Vici, Giovanni Scognamiglio, Daniele Generali, Alberto Zambelli, Marco Colleoni, Corrado Tinterri, Francesco Scanzi, Leonardo Vigna, Paola Scavina, Teresa Gamucci, Emilia Marrazzo, Angelo Fedele Scinto, Rossana Berardi, Maria Agnese Fabbri, Graziella Pinotti, Daniela Franco, Daniela Andreina Terribile, Giuseppe Tonini, Daniela Cianniello, Sandro Barni

**Affiliations:** 1 Università La Sapienza di Roma, Dipartimento Medicina Clinica e Molecolare, Rome, Italy; 2 Policlinico Universitario Agostino Gemelli, IRCCS, Roma, Italy; 3 IRCCS Regina Elena National Cancer Institute, Rome, Italy; 4 Fondazione IRCCS Istituto Nazionale dei Tumori, Milano, Italy; 5 Policlinico Sant’Orsola Malpighi, Bologna, Italy; 6 Ospedale San Raffaele, Milano, Italy; 7 Ospedale Gaetano Bernabeo, Ortona, Italy; 8 Policlinico Umberto I, Roma, Italy; 9 Azienda Ospedaliera Universitaria Federico II, Napoli, Italy; 10 ASST Spedali Civili, Brescia, Italy; 11 IRCCS Regina Elena National Cancer Institute, Roma, Italy; 12 Ospedale Valduce, Como, Italy; 13 ASST di Cremona, Cremona, Italy; 14 ASST Papa Giovanni XXIII, Bergamo, Italy; 15 Istituto Europeo di Oncologia, Milano, Italy; 16 Istituto Clinico Humanitas, Rozzano, Italy; 17 IRCCS Multimedica, Sesto San Giovanni, Italy; 18 Azienda Ospedaliera San Camillo Forlanini, Roma, Italy; 19 Azienda Ospedaliera San Giovanni - Addolorata, Roma, Italy; 20 Ospedale SS. Trinità, Sora, Italy; 21 Istituto Clinico Humanitas, Rozzano, Italy; 22 Ospedale San Giovani Calibita Fatebenefratelli, Roma, Italy; 23 Azienda Ospedaliero Universitaria Ospedali Riuniti di Ancona, Torrette, Italy; 24 Ospedale di Belcolle, Viterbo, Italy; 25 Ospedale di Circolo e Fondazione Macchi, Varese, Italy; 26 Ospedale Nuovo Regina Margherita, Roma, Italy; 27 Policlinico Universitario Campus Biomedico, Roma, Italy; 28 Istituto Nazionale Tumori Fondazione G. Pascale, Napoli, Italy; 29 ASST BG Ovest Ospedale Treviglio, Treviglio, BG Italy

**Keywords:** Predictive markers, Breast cancer, Cancer genomics, Breast cancer

## Abstract

Clinicopathological prognostic features have limited value to identify with precision newly diagnosed patients with hormone receptor (HR)-positive, HER2-negative breast cancer (BC), who would benefit from chemotherapy (CT) in addition to adjuvant hormonal therapy (HT). The 21-gene Oncotype DX Breast Recurrence Score^®^ (RS) assay has been demonstrated to predict CT benefit, hence supporting personalized decisions on adjuvant CT. The multicenter, prospective, observational study PONDx investigated the real-life use of RS^®^ results in Italy and its impact on treatment decisions. Physicians’ treatment recommendations (HT ± CT) were documented before and after availability of RS results, and changes in recommendations were determined. In the HR+ HER2− early BC population studied (*N* = 1738), physicians recommended CT + HT in 49% of patients pre-RS. RS-guided treatment decisions resulted in 36% reduction of CT recommendations. PONDx confirms that RS results provide clinically relevant information for CT recommendation in early-stage BC, resulting in a reduction of more than a third of CT use.

## Introduction

Breast cancer (BC) is the most common type of malignancy in women and the second most common cancer overall. According to the GLOBOCAN database, the age-standardized incidence rate in Italy is 92.8 cases per 100,000 women per year^1^.

Classification of BC is primarily based on the expression of key signaling molecules including receptors for the female sex hormones (HR) estrogen and progesterone (ER, PR), as well as the human epidermal growth factor 2 (HER2).

For the large population of patients with HR+ (ER+ and/or PR+), HER2− tumor status (~70% of nonmetastatic primary BCs)^2^, adjuvant hormonal therapy (HT) is recommended and is used as the standard treatment for most patients. When adjuvant chemotherapy (CT) is given in addition to HT, the side effects and risk burden require adequate measures to best identify the patients who will most likely derive a clinical benefit.

Traditionally, a set of clinical and pathological features have been used to evaluate the prognosis of the patient and guide decisions on adjuvant therapies. Besides the HR status, prognostic parameters include patient age, lymph node involvement, tumor size, histological type, and grade, as well as the Ki67 proliferation marker^3^. However, these conventional clinical and histopathological markers have insufficient specificity and sensitivity to precisely predict which patients are likely to experience a significant benefit of CT on cancer recurrence that outweighs the substantial side effects.

Conventional parameters apparently have limited association with tumor biology, leaving a broad margin of predictive uncertainty^4^. Accordingly, only a minority of HR+, HER2− patients appears to benefit from CT: in a large meta-analysis performed by the Early Breast Cancer Trialists’ Collaborative Group, the reduction of the 10-year recurrence rate by adjuvant CT was <10%^5^. Recently, the prospective ECOG-ACRIN Trial Assigning Individualized Options for Treatment (TAILORx) established in over 10,000 patients that the vast majority of HR+, HER2−, node-negative (N0) primary BC patients (about to 80%) do not derive benefit from CT in terms of recurrence risk^6^.

The 21-gene Oncotype DX Breast Recurrence Score multigene assay was developed to aid physicians in making personalized CT treatment decisions in HR+, HER2− early-stage BC patients. Clinical validation and utility of the assay have been demonstrated in multiple studies with >96,000 N0 and node-positive (N+) BC patients worldwide^6–12^.

Two studies have validated the prediction of a CT benefit by the Oncotype DX^®^ test for N0 patients with level 1B and 1A evidence. NSABP B-20^13^ was a prospective analysis of archived, preserved samples and it demonstrated that patients with Recurrence Score (RS) results of 26–100 derive a substantial benefit from CT, whereas patients with an RS 0–10 had excellent clinical outcomes at 9 years with endocrine therapy alone^14^. The TAILORx study prospectively assessed in a large, randomized population the merits of CT in patients with RS 11–25 and demonstrated that, overall, they did not derive a significant benefit from CT. Taken together, these results established that the Oncotype DX assay can guide CT treatment decisions such that N0 patients with RS 0–25 can safely forego CT, whereas patients with RS 26–100 do derive substantial benefit from CT used in addition to hormonal treatment. In postmenopausal women with N+, ER + BC, the prospective, retrospective analysis of SWOG-8814 trial established that N1 patients with RS 0–17 could be safely spared CT, whereas patients with RS 31–100 achieved a strong clinical benefit with CT^15^.

For N0 and N+ disease, aggregate data from prospective registries with 5–10 years of observation confirm that the RS result consistently identifies patients with good clinical outcomes when treated with HT alone^10,16,17^.

The Oncotype DX assay has been incorporated into clinical and pathological guidelines of major international medical societies including the European Society of Medical Oncology, St. Gallen Consensus Conference, American Society of Clinical Oncology, and American Joint Committee on Cancer. The National Comprehensive Cancer Network Guidelines state that the Oncotype DX assay is the only test with proven validity to predict CT benefit^18^.

In decision impact studies in Europe conducted before publication of the TAILORx study and, hence, before the predictive cutoffs were established with precision, outcomes have shown the utility of the RS result in clinical practice. In a meta-analysis of more than 500 N0, HR+, HER2− primary BC patients from four studies, the overall rate of recommendation change was 32% post- vs. pre-testing, whereas the CT recommendation rate decreased from 55% to 34%^19^.

Here we describe the results of the multicenter, prospective, observational study, PONDx, which was performed in Italy from February 2016 to December 2017, and which investigated the real-life use of the Oncotype DX Breast Recurrence Score test by physicians treating early BC patients in routine care in clinical BC reference centers. The study primarily evaluated the impact of the Oncotype DX assay on physicians’ treatment decisions. A further objective was the characterization of the patient population in which the test is used in real-life settings at clinical BC reference centers in Italy.

## Results

Data from 1738 BC patients who underwent Oncotype DX testing were available from 27 reference centers located in 6 regions of Italy (Lombardia, Lazio, Emilia Romagna, Campania, Abruzzo, and Marche). In the present analysis, 14 patients were excluded due to incomplete data, leaving 1724 in the analysis cohort.

The tumors diagnosed in the participating patients were mostly invasive ductal HR-positive carcinomas, with histological grade 2 and 3, and tumor size ranging from 1 to 5 cm, with Ki67 expression mostly in the range from 10% to >30%. The majority of patients were >50 years old; 36% were premenopausal and 55% postmenopausal (Table 1).

**TABLE 1:** Patient and tumor characteristics in the overall population (*N* = 1738).

Parameter	Characteristics	Number of patients	Percentage of patients
Age	<35 Years	26	1%
	35–50 Years	655	38%
	51–70 Years	831	48%
	>70 Years	226	13%
Gender	Female	1720	99%
	Male	18	1%
Menopausal status	Pre	623	36%
	Peri	137	8%
	Post	960	55%
	ND	18	1%
Histological subtype	Other	110	6%
	Ductal	1417	82%
	Lobular	211	12%
Histological grade	G1	165	9%
	G2	1090	63%
	G3	483	28%
Tumor size	<1 cm	232	13%
	1–2 cm	1052	61%
	2.1–5 cm	432	25%
	>5 cm	22	1%
Nodal status	N0	1192	69%
	Nmic	113	7%
	N1	433	25%
ER status	Negative	5	<1%
	Positive	1733	>99%
PR status	Negative	164	9%
	Positive	1574	91%
HER2 status	Equivocal	72	4%
	Negative	1637	94%
	Positive	29	2%
Ki67 expression	<10%	169	10%
	10–20%	576	33%
	21–30%	611	35%
	>30%	377	22%
	ND	5	<1%

*ND* not determined.

The distribution of Recurrence Score results is shown in Table 2 (left columns). Judged by the conventional cut points, 57% of the overall population were in the RS 0–17, 34% in the RS 18–30 and 9% in the RS 31–100 group. Using the TAILORx-based cut points, 83% of the population was in the RS 0–25 group and 17% in the RS 26–100 group.

**TABLE 2:** Oncotype DX Breast Recurrence Score® results categorized according to RS groups used prior to TAILORx (left) and RS groups based on TAILORx cut points (shaded columns, right).

	RS categories as defined prior to TAILORx			RS categories based on TAILORx cut points	
	0–17	18–30	31–100	0–25	26–100
Overall population (*N* = 1738)	987 (57%)	588 (34%)	163 (9%)	1444 (83%)	294 (17%)
Histological grade					
G1 (*n* = 165)	123 (75%)	41 (25%)	0 (0%)	164 (99%)	1 (1%)
G2 (*n* = 1090)	684 (63%)	351 (32%)	196 (5%)	969 (89%)	121 (11%)
G3 (*n* = 483)	179 (37%)	196 (41%)	108 (22%)	311 (64%)	172 (36%)
Tumor size					
<1 cm (*n* = 232)	144 (62%)	74 (32%)	14 (6%)	195 (84%)	37 (16%)
1–2 cm (*n* = 1052)	589 (56%)	369 (35%)	94 (9%)	888 (84%)	164 (16%)
2.1–5 cm (*n* = 432)	240 (56%)	138 (32%)	54 (13%)	343 (79%)	89 (21%)
>5.0 cm (*n* = 22)	14 (64%)	7 (32%)	1 (5%)	18 (82%)	4 (18%)
Nodal status					
N0 (*n* = 1192)	644 (54%)	417 (35%)	131 (11%)	959 (80%)	233 (20%)
Nmic (*n* = 113)	61 (54%)	44 (39%)	8 (7%)	97 (86%)	16 (14%)
N1 (*n* = 433)	282 (65%)	127 (29%)	24 (6%)	388 (90%)	45 (10%)
Ki67 expression					
<10% (*n* = 168)	127 (75%)	35 (21%)	7 (4%)	162 (96%)	7 (4%)
10–20% (*n* = 576)	379 (66%)	179 (31%)	18 (3%)	524 (91%)	52 (9%)
21–30% (*n* = 611)	337 (55%)	227 (37%)	47 (8%)	508 (83%)	103 (17%)
>30% (*n* = 377)	142 (38%)	146 (39%)	89 (24%)	247 (66%)	130 (34%)
Histology					
Lobular	144 (67%)	61 (29%)	9 (4%)	NA	NA
Patient age					
<35 Years (*n* = 26)	10 (38%)	12 (46%)	4 (15%)	19 (73%)	7 (27%)
35–50 Years (*n* = 655)	403 (62%)	201 (31%)	51 (8%)	566 (86%)	89 (14%)
51–70 Years (*n* = 831)	443 (53%)	304 (37%)	84 (10%)	675 (81%)	156 (19%)
>70 Years (*n* = 226)	131 (58%)	71 (31%)	24 (11%)	184 (81%)	42 (19%)
Menopausal status					
Pre (*n* = 623)	378 (61%)	196 (31%)	49 (8%)	535 (84%)	88 (16%)
Peri (*n* = 137)	75 (55%)	48 (35%)	14 (10%)	116 (85%)	21 (15%)
Post (*n* = 960)	522 (54%)	339 (35%)	99 (10%)	777 (81%)	183 (19%)

It is interesting to note the discordance of some of the key classical pathological parameters and RS results. Using the TAILORx-based RS cut points (Table 2, right columns), the discordance between some classical pathological parameters and RS result remains pronounced, with a significant proportion of grade 2 (89%) and grade 3 (64%) patients having RS 0–25, indicating no CT benefit. Similarly, patients with the lowest (<10%) or highest (>30%) level of expression of the proliferation marker Ki67 were found to have RS 26–100 and RS 0–25, respectively. The latter may in part be related to the known lack of reproducibility of Ki67 assays^20–22^, yet could be expected considering Ki67 has not been demonstrated to correlate with CT response^23^. No discernible correlations were found between RS result and tumor size, age, or menopausal status.

In the population analyzed for treatment recommendation (*n* = 1683), the physicians recommended CT + HT in 824 patients (49%) prior to the availability of the RS result, whereas 859 (51%) were assigned to HT alone. Patients with recommendations for other therapies than CT or CT + HT were excluded from this analysis.

**Fig. 1:**
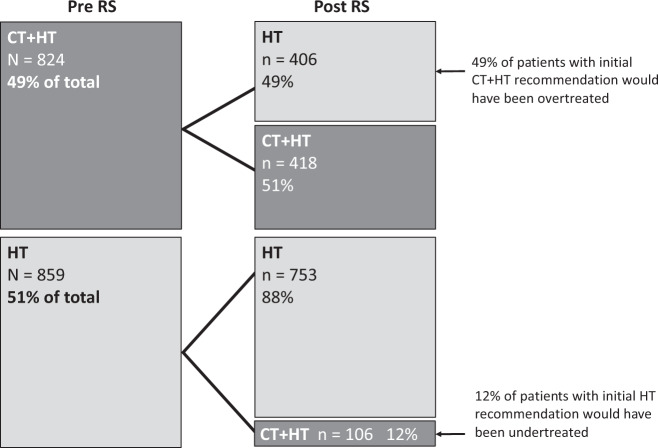
Changes in treatment recommendations before and after availability of the Recurrence Score result. Rates of hormone therapy alone (HT) or chemo-endocrine therapy (CT+HT) recommendations before testing (Pre RS) and changes in recommendations based on the test resutls (Post RS).

After the RS results became available, the physicians changed their decision in 512 patients (30%). Consequently, the number of patients with a CT + HT recommendation dropped from 824 to 524, corresponding to a net reduction of CT recommendations of 36% (Table 3 and Fig. 1).

**TABLE 3:** Recommendations by the treating physician regarding adjuvant anti-tumor therapy before (PRE-RS) and after availability (POST-RS) of the Recurrence Score result. For these analyses, patients with recommendations other than CT + HT or HT were excluded.

		Treatment recommendations, *n* (%)				Change in CT + HT recommendations (%)
		PRE-RS		POST-RS		
Population		CT + HT	HT	CT + HT	HT	
Overall population	*N* = 1683	824 (49%)	859 (51%)	524 (31%)	1159 (69%)	−36%
N0	*n* = 1160	512 (44%)	648 (56%)	374 (32%)	786 (68%)	−27%
Nmic	*n* = 109	54 (50%)	55 (50%)	33 (30%)	76 (70%)	−39%
N1	*n* = 414	258 (62%)	156 (38%)	110 (28%)	297 (72%)	−55%
Grade 3	*n* = 475	350 (74%)	125 (26%)	254 (53%)	221 (47%)	−27%
Ki67 > 20%	*n* = 962	608 (63%)	354 (37%)	386 (40%)	576 (60%)	−37%
Lobular breast cancer	*n* = 203	98 (52%)	105 48%)	46 (23%)	157 (77%)	−53%
Age > 50 years	*n* = 1027	464 (45%)	563 (55%)	314 (31%)	713 (69%)	−31%
Age ≤ 50 years	*n* = 656	360 (55%)	296 (45%)	210 (32%)	446 (68%)	−42%

Looking at selected groups defined by tumor characteristics, the following picture emerges among the patients with clinicopathological high-risk tumors (Table 3): in patients with grade 3 malignancies (*n* = 475), physicians changed their treatment recommendation in 37% of cases, leading to a 27% net reduction of CT + HT. Patients with Ki67 expression >20% (*n* = 962) had their recommendation amended in 36%, with a 37% net reduction in CT + HT recommendations (from 608 to 386 patients).

For patients with N0 nodal status (*n* = 1160) CT + HT recommendations declined by 27% (from 512 to 374 patients). In contrast, the group with N1 disease (*n* = 414) saw the frequency of CT + HT recommendations reduced by a net percentage of 55% after the RS results became available (from 258 to 110 patients). Similarly, pronounced effects were observed for the subset of patients with Nmic (*n* = 109) and with lobular BC (*n* = 206). In these specific groups, the net reduction of CT + HT was 39% and 53%, respectively.

Regarding patients aged >50 vs. ≤50 years, both age groups showed a strong net reduction of CT + HT recommendations (by 31% and 42%, respectively). In the group with initial recommendations of HT-only (*N* = 859), a minor fraction of patients had their recommended treatment changed to CT + HT (*n* = 102; 12%).

**Fig. 2:**
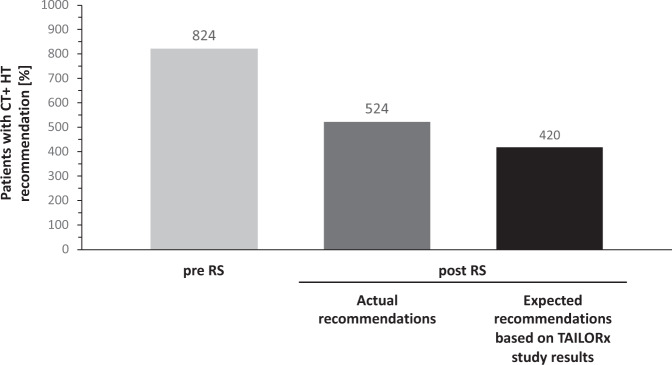
Patients with post-RS recommendations for chemo-endocriine therapy (CT + HT): actual number based on previous RS cut points and expected percentage assuming decision-making according to TAILORx results (N = 1683). Pre RS: treatment recommendations before avalability of the Recurrence Score result. Post RS: treatment recommendations accounting for the Recurrence Score result.

A simulation of expected treatment recommendations after availability of the RS result was performed based on TAILORx RS cut points^6^ and the estimated interpretation of the RS results according to the findings of the TAILORx study (see “Methods” and Table 4). According to these premises, 75% (*n* = 1263) of patients would receive HT-only and 25% (*n* = 420) adjuvant CT + HT regimens in this setting, corresponding to a relative reduction of 49% for the overall population (Fig. 2) (47% for N0) (Table 5). Regarding the age groups >50 vs. ≤50 years, there was a net reduction of CT + HT recommendations (by 41% and 50%, respectively). The proportion of patients ≤50 years with N0 disease and RS 16–20 and RS 21–25 represent, respectively, 9% and 5% of the overall N0 population.

**TABLE 4:** Algorithm used to estimate distribution of post-RS treatment recommendations in N0 patients based on TAILORx results.

Nodal status	Patient age	Treatment recommendations			
		RS 0–15	RS 16–20	RS 21–25	RS 26–100
N0	≤50 Years	HT-only	HT-only 90%	HT-only 60%	CT + HT
			CT + HT 10%	CT + HT 40%	
	>50 Years	HT-only			CT + HT

**TABLE 5:** Expected recommendations assuming decision-making according to TAILORx results.

Population		Treatment recommendations, *n* (%)				Change in CT + HT recommendations (%)
		PRE-RS		POST-RS		
		CT + HT	HT	CT + HT	HT	
Overall population	*N* = 1683	824 (49%)	859 (51%)	420 (25%)	1263 (75%)	−49%
N0	*N* = 1160	512 (44%)	648 (56%)	270 (23%)	890 (77%)	−47%

## Discussion

In this observational study, we documented the use of the Oncotype DX assay and its impact on physicians’ therapeutic recommendations in a sizeable patient population from 27 BC reference centers in 6 regions of Italy. Our data add to the growing body of evidence from RS result decision impact studies with N0 and N+ tumors performed in Europe^19,24–26^, North America^27,28^, or Australia^29^.

Assessment of the recurrence risk by the Oncotype DX Breast Recurrence Score assay influenced physician’s choice of adjuvant regimens, resulting in an overall reduction of 36% in CT recommendations vs. decisions based on prognostic-only clinicopathological risk parameters. Recalculation of the expected post-RS recommendations using estimates based on TAILORx cut points and results showed a higher reduction (49%) of the proportion of patients recommended CT in the total population. This observation was consistent in both age groups of ≤50 and >50 years.

The significant overall reduction in CT recommendations guided by RS results in this study correlates with that of other studies published worldwide, reporting up to 47% reduction with similar patient populations and pre-TAILORx cut points. This indicates a consistent decision impact of the RS results^19,24–29^. The relative reduction in CT recommendations is relevantly influenced by clinical practice and baseline CT usage. This has been reported to be highly variable across countries, centers, and within single centers^4^.

The findings of our study support previous evidence^4^ demonstrating physicians’ uncertainty to recommend adjuvant treatment relying on prognostic-only factors that have not been correlated with the prediction of CT benefit. Tumor grade and size, in particular, are not stringently related to tumor biology. Although they estimate patient prognosis, i.e., risk of recurrence, they do not predict response to CT as a specific treatment option^23^.

In addition, the high variability and lack of standardization on Ki67 or histological tumor-grade assessments leave the treating physicians with a margin of uncertainty^22^. The Oncotype DX test results can provide confidence for CT treatment decisions with a strong body of evidence on the prediction of CT benefit^1^. Of note, no patient subgroup in this study was identified, which could forego Oncotype DX RS testing without losing potentially useful predictive information on CT benefit or lack thereof.

Analysis of the widely used Ki67 proliferation marker revealed that 66% of the patients with high Ki67 expression (>30%) had an RS result 0–25 and would not be expected to derive benefit from CT, indicating that Ki67 should probably not be used as a dominant indicator for treatment decisions. Similar considerations apply for patients with grade 3 tumors, 64% of whom had an RS result in the 0–25 range. This is consistent with observations in the TAILORx study where 73% of patients with a high clinical risk based on tumor-grade and size assessment had an RS result 0–25 and hence might have been overtreated if the RS result had not been used for the treatment decision^6^.

Conversely, a minor fraction of patients had their initial HT-only recommendations changed to actually receive additional CT, suggesting that they were rescued from potential undertreatment based on conventional criteria.

The overall change in CT recommendations by Oncotype DX RS results in this study, based on pre-TAILORx cut points, is in the range of that in other published studies with similar patient populations worldwide, indicating a consistent interpretation of the RS results^19,24–29^. The strength of the present study lies in its size: this is the largest decision impact study reported to date. Furthermore, this real-life study confirms the results of a randomized controlled trial. Its main limitation is that the study was performed before the availability of TAILORx data, demonstrating that even a higher proportion of patients could be spared CT, thanks to the RS results. Consequently, we might underestimate the net CT sparing effect of the Oncotype DX test. Although the results of the TAILORx study were practice-changing for N0 patients, the ongoing RxPONDER trial will provide additional information on the clinical usefulness of the Oncotype DX assay in women with HR-positive BC and positive axillary nodes. In this study, we observe a very significant reduction of CT recommendations (55%) for patients with limited nodal involvement (N1). This is supported by the consistent evidence from the SWOG-8814^15^ and Plan B^8^ studies, as well as prospective registries^9,11^ supporting CT sparing for patients with the lowest RS results.

Chemotherapeutic regimens used in the adjuvant setting in women with early BC are associated with a significant risk of acute and long-term adverse effects—the latter including fatigue, cardiotoxicity, cognitive impairment^30^, peripheral neuropathy, and cases of secondary malignancies including leukemia. Quality of life and working ability may be reduced at least temporarily by these treatments as well. Therefore, tools refining the population that derives appropriate benefit to justify the adverse effects of adjuvant CT serve an important medical need in patients with HR+, HER2− primary BC. The Oncotype DX assay is such an instrument and may contribute significantly to a reduction in the use of CT for patients who are unlikely to derive benefit. Conversely, the assay allows identification of a group of 15–20% of HR+, HER2− early BC patients who derive a substantial benefit from CT. In these patients, CT in addition to HT consistently results in lower distant recurrence rates than HT alone^5,13,14^.

In our study, we reported, overall, 83% of patients had RS 0–25 guiding towards a significant de-escalation of CT. These proportions are consistent with the TAILORx study and with patient registries such as the Surveillance, Epidemiology and End Results registry in the United States^31^ and the Clalit registry in Israel^10^, reporting 84% and 80% patients with RS 0–25, respectively. This majority of patients (about 80%) with RS 0–25 consistently showed excellent clinical outcome with HT alone and hence can safely be spared CT.

Results from the TAILORx study are considered practice-changing, because for the first time it was shown in a large prospective randomized trial that a sizeable group of patients could be identified with a unique multigene assay to derive minimal or no benefit from CT. For patients initially recommended to receive chemo-HT in our study, use of the Oncotype DX test with TAILORx cut points led to a reduction by nearly 50% in CT recommendations.

In addition to the clinical benefit to the patients who are spared adverse effects, reduction of CT use has relevant implications for the healthcare system through reduction of direct expenses (cytotoxic drugs and their application) and indirect costs (managing side effects). Benefits to healthcare-associated and societal costs importantly include diminished duration of absence from work, which has been shown to be significantly prolonged by a median of 7 months for patients who receive CT. In fact, the use of CT was one of the factors with the highest risk ratio of delayed time to work after primary BC^32^, responsible for more than a quarter of the total costs of CT^33^. Other genomic assays are available for early BC patients; however, it is noteworthy that although Oncotype DX brings value guiding CT decisions based on direct evidence of prediction of CT benefit, other genomic assays are prognostic-only. Decision impact studies with MammaPrint^®^ prognostic assay^34,35^, EndoPredict^®^^36^, or Prosigna^®^^37,38^ consistently reported a limited impact of net CT use related to a balance between significant reduction from CT-HT to CT and a significant increase from HT alone to CT-HT.

The observations on the use and impact of the Oncotype DX Breast Recurrence Score test on the participating reference centers of PONDx in Italy support the notion that the test provides clinically useful predictive information, complementing standard clinical and pathological risk parameters for patients with HR+, HER2− N0/N1 primary BC. The physicians used the results to modify their original treatment recommendations, which resulted in a reduction of patients recommended for CT by more than a third.

Estimating the effects based on the RS categories and outcomes established in the recently completed large prospective TAILORx trial confirmed the primary results of PONDx, indicating the potential for an even more pronounced reduction of CT recommendations and thereby potentially sparing a significant proportion of patients from acute and long-term toxicities of these treatments.

## Methods

### Patients eligibility

Eligible patients fulfilled the validated criteria for use of the Oncotype DX assay: patients aged ≥18 years with a recent diagnosis of early, single-invasive ER+ HER2− BC and available information on lymph node involvement categorized as N0, Nmic (micrometastatic node involvement), or N1 (one to three positive nodes).

Baseline patient documentation included age and sex, menopausal status, conventional clinical and pathological tumor characteristics including histologic type (lobular/ductal), tumor size and grade, nodal status, receptor status (ER, PR, HER2), Ki67 expression, and RS results as soon as available.

### Ethics

Patients provided written informed consent before participation in the study. The protocol was approved by the Ethics Committee of all participating institutions: Università La Sapienza di Roma, Policlinico Universitario Agostino Gemelli, IRCCS Regina Elena National Cancer Institute, Fondazione IRCCS Istituto Nazionale dei Tumori, Policlinico Sant’Orsola Malpighi, Ospedale San Raffaele, Ospedale Gaetano Bernabeo, Policlinico Umberto I, Azienda Ospedaliera Universitaria Federico II, ASST Spedali Civili, IRCCS Regina Elena National Cancer Institute, Ospedale Valduce, ASST di Cremona, ASST Papa Giovanni XXIII, Istituto Europeo di Oncologia, Istituto Clinico Humanitas, IRCCS Multimedica Sesto San Giovanni, Azienda Ospedaliera San Camillo Forlanini, Azienda Ospedaliera San Giovanni - Addolorata, Ospedale SS. Trinità, Istituto Clinico Humanitas, Ospedale San Giovani Calibita Fatebenefratelli, Azienda Ospedaliero Universitaria Ospedali Riuniti di Ancona, Ospedale di Belcolle, Ospedale di Circolo e Fondazione Macchi, Ospedale Nuovo Regina Margherita, Policlinico Universitario Campus Biomedico, Istituto Nazionale Tumori Fondazione G. Pascale, and ASST BG Ovest Ospedale Treviglio.

### Treatment decisions

Prior to Oncotype DX testing, CT was recommended to patients with worse prognosis based on clinical, pathological, and biological features as per the local clinical practice. Individual treatment modalities (HT, CT-HT) recommended by the treating physician were documented before (pre-RS) and after (post-RS) availability of the test results. Patients followed recommendations that emerged post-RS result.

Descriptive analyses were performed for the overall population and subpopulations of patients with clinical high-risk tumors defined by grade 3 disease and/or >20% Ki67 positivity, patients with N0 vs. N1 nodal status, and those with cancers of lobular histology. For these populations, changes in treatment recommendations regarding HT and/or CT were determined by comparing the percentage of patients receiving a recommendation of HT or HT + CT before vs. after the test results became available to the treating physician. The primary analysis used the RS cut points commonly used before TAILORx was published, to define three RS groups: 0–17, 18–30, and 31–100.

An additional exploratory analysis regarding the influence of the RS result on treatment recommendations was performed using the RS cut points for N0 patients defined by the TAILORx trial and their expected interpretation in clinical practice. In the TAILORx study, exploratory analyses suggested that all N0 patients above the age of 50 years with RS 0–25 have no CT benefit. For younger patients (≤50 years), an RS of 0–15 indicated no CT benefit, whereas some CT benefit was derived for RS 16–20 (1.6%) and RS 21–25 (6.5%). Patients with N1 disease were assigned to RS groups according to previous cut points as described above. Thus, the algorithm described in Table 4 was recommended by an expert panel based on the finding from exploratory analyses of TAILORx, suggesting a potentially clinically meaningful benefit from CT for a small number of patients, and was used for patients with nodal status N0 in the analysis of presumed treatment recommendations based on TAILORx findings.

### Reporting summary

Further information on research design is available in the Nature Research Reporting Summary linked to this article.

## Supplementary information

reporting summary

## Data Availability

The datasets that support the findings of this study will be made available upon reasonable request from the corresponding author, Dr. Francesco Cognetti, email address: francesco.cognetti@ifo.gov.it. The data generated and analyzed during this study are described in the following metadata record^39^: 10.6084/m9.figshare.13049816.
